# Prelabour caesarean section and neurodevelopmental outcome at 4 and 12 months of age: an observational study

**DOI:** 10.1186/s12884-020-03253-8

**Published:** 2020-09-25

**Authors:** Mehreen Zaigham, Lena Hellström-Westas, Magnus Domellöf, Ola Andersson

**Affiliations:** 1 Department of Obstetrics & Gynaecology, Lund University and Skåne University Hospital, 205 01 Malmö, Sweden; 2 Department of Women’s and Children’s Health, Uppsala University, Uppsala, Sweden; 3 Department of Clinical Sciences, Pediatrics, Umeå University, Umeå, Sweden; 4 Department of Clinical Sciences Lund, Pediatrics, Lund University, Lund, Sweden

**Keywords:** Prelabour caesarean section, Elective caesarean, Cesarean, Vaginal delivery, Birth, Neurodevelopmental outcome, Developmental skills, Short-term outcome, Cord clamping

## Abstract

**Background:**

With prelabour caesarean section rates growing globally, there is direct and indirect evidence of negative cognitive outcomes in childhood. The objective of this study was to assess the short-term neurodevelopmental outcomes after prelabour caesarean section as compared to vaginally born infants.

**Methods:**

We conducted a prospective, observational study of infants delivered by prelabour caesarean section at the Hospital of Halland, Halmstad, Sweden and compared their development with an historical group of infants born by non-instrumental vaginal delivery.

**Results:**

Infants born by prelabour caesarean section were compared with a group of vaginally born infants. Follow-up assessments were performed at 4 and 12 months. Prelabour caesarean infants (*n* = 66) had significantly lower Ages and Stages Questionnaire, second edition (ASQ-II) scores in all domains (communication, gross motor, fine motor, problem solving and personal-social) at 4 months of age with an adjusted mean difference (95% CI) of − 20.7 (− 28.7 to − 12.6) in ASQ-II total score as compared to vaginally born infants (*n* = 352). These differences remained for gross-motor skills at the 12 month assessment, adjusted mean difference (95% CI) -4.7 (− 8.8 to − 0.7), *n* = 62 and 336.

**Conclusions:**

Adverse neurodevelopmental outcomes in infants born by prelabour caesarean section may be apparent already a few months after birth. Additional studies are warranted to explore this relationship further.

## Background

Caesarean sections are effective and lifesaving procedures but the World Health Organization (WHO) regards rates higher than 10% as not associated with reductions in maternal and newborn mortality rates. Sweden has one of the lowest caesarean section (CS) rates in the world (17%). On the other end of the spectrum, countries like Chile, Mexico and Turkey have rates ranging from 45 to 53% [1]. The rising rate of caesarean births across the world has been directly and indirectly associated with negative child cognitive outcomes. Caesarean births have been associated with asthma, type 1 diabetes, allergies [2–4], obesity [5] and have been linked to impaired functioning and lower academic performance [6, 7]. Recent studies have shown that prelabour CS may be associated with adverse child cognitive score outcome [8] and school performance [9]. Furthermore, caesarean sections have been associated with altered mother-child interactions [10] and lower rates of breastfeeding [11]. A large population-based study in Sweden [12] found that children delivered by CS were at higher risk to be treated in hospital, all diagnoses considered, in their pre-school years as compared to non-instrumental vaginally delivered children.

Our research group has shown that waiting to clamp the umbilical cord for 30 s after prelabour CS resulted in higher iron stores at 4 months of age as compared to early cord clamping (CC) after vaginal birth [13]. In fact, the effect on iron stores was as pronounced as when clamping the umbilical cord after 180 s with vaginal delivery (delayed CC) [13]. This would indicate that waiting for 30 s during prelabour caesarean birth to clamp the cord would suffice to ensure sufficient placental transfusion to ensure enhanced iron stores at 4 months. In research on delayed cord clamping in vaginal births, improved brain myelin content is present at 4 and 12 months [14, 15], but neurodevelopment assessed by Ages & Stages Questionnaire, 2nd edition (ASQ-II) was not associated to cord clamping time, placental transfusion or iron stores at 4 and 12 months [16, 17]. After additional growth and maturation, improved fine motor function and to some extent social behaviour was shown among children born after delayed CC ≥ 180 s [18].

The aim of this study was to investigate the association between prelabour CS and neurodevelopmental outcomes in infants at 4 and 12 months of age. This was an observational study, comparing a group of children born with scheduled CS with a control group of vaginally born children.

## Methods

We conducted a prospective, observational study of infants delivered by prelabour CS at the Hospital of Halland, Halmstad, Sweden. Comparisons were made to reference data from a study of vaginal births in which neonates were randomized to delayed CC (≥180 s) or early CC (≤30 s) with similar inclusion and exclusion criteria. We decided to merge the vaginal delivery group together (delayed CC and early CC) based on previous evidence that cord clamping time was not associated with differences in neurodevelopment assessed by ASQ-II at 4 and 12 months of age [13, 17]. The clinical trial was approved by the Regional Ethical Review Board of Lund University (2008/41 and 2009/344).

From June 6, 2010 to February 29, 2012, designated midwives approached women planned for CS and informed them of the study. Written informed consent was obtained from all participants and both parents were required to sign consent forms in order to participate in the study. The historical control group consisted of 338 term neonates born by non-instrumental vaginal delivery and were included between April 16, 2008 and May 22, 2009. The results from the trial have been reported elsewhere [16–20].

In short, pregnant women were eligible for participation if they met the following criteria: non-smoking, normal pregnancy (normal defined as the absence of preeclampsia, diabetes, prolonged rupture of membranes or other signs of infection) and term pregnancy (gestational age ranging from 37 + 0 to 41 + 6 weeks). Adequate proficiency in Swedish was also a prerequisite to participate in the study. Of the exclusion criteria, serious congenital malformations, syndromes or other congenital diseases that could possibly affect the outcomes, all such cases were excluded from the study. An additional eligibility criterion for the prelabour CS group was admission for a scheduled CS. No emergency CS was included in the study.

According to local practice, directly after birth, newborns in the prelabour CS group were placed on the mother’s thighs or beside her on the operation table whilst obstetricians waited 30 s to clamp the umbilical cord. The exact timing of cord clamping after 30 s was chosen by the obstetric board at the hospital before initiation of the current study. The timing of the clamping was noted. After clamping, blood samples for blood gas evaluation were taken from the placental side of the umbilical vessels.

Relevant neonatal data such as Apgar score, birth weight, length and head circumference were recorded according to local routines. An assessment of the newborn’s well-being was carried out by a midwife at 1 and 6 h after birth. Data was prospectively collected in the study protocol and it was noted if the newborn had any respiratory difficulties (presence of nostril flaring, grunting, respiratory frequency above 60 breaths/minute and intercostal retractions) including information about breastfeeding. Neither the mother nor the midwife performing the intervention could be blinded, but all staff involved in collection or analyses of ASQ-II were blinded to the allocation group. Infants were scheduled for follow-up visits at 4 and 12 months of age.

### Outcomes

The primary outcome of this observational study was neurodevelopment at the 4 month and 12 month follow-up assessed by the ASQ-II (ASQ-II total score and scores on 5 subdomains: communication, gross motor, fine motor, problem solving, and personal-social).

### Ages and stages questionnaire

Parent-reported infant development was assessed using the ASQ-II [21]. Parents were sent home forms 1 month prior to the planned assessments. For use in the study, the ASQ-II 4 and 12 month questionnaires were translated in to Swedish. Prior permission was obtained from Paul H. Brookes Publishing Company. Cut-off scores were created according to the ASQ-II manual, i.e. by subtracting the mean score with 2 SDs from the results in the vaginally born group [18, 20]. In addition, scores were adjusted according to the ASQ-II manual if the ASQ-IIs were not completely answered.

### Statistical analysis

Sample size in the vaginally delivered group was fixed as data from a former study was used [16]. The sample size for the prelabour CS group was initially chosen to find a difference in Ferritin at 4 months of age [13]. With this sample size, a difference in ASQ-II total score of ≥10.5 at 4 months and ≥ 15.2 at 12 months would be significant according to a post hoc power analysis. Comparisons between groups were analysed using two-sided Student *t* test, Mann-Whitney *U* test, or the Fisher exact test where appropriate. The data software used for the study was SPSS for IBM, version 25.0 (SPSS Inc). Relative risks and 95% confidence intervals (CI) were calculated by the web-based JavaStat calculator [22]. Spearman rank correlation coefficient (ρ) was determined to assess maternal and neonatal variables that were associated with outcomes at 4 and 12 months (ASQ-II total score), Table 1. Outcomes were entered into a linear regression model when the variables had a correlation significance of *P* < 0.10. The variables were then analysed by stepwise backward selection, leaving only variables with *P* < 0.05 in the model Table 2.

**Table 1:** Maternal and infant characteristics after prelabour caesarean section (CS) or vaginal delivery and correlations between these characteristics, intervention group and Ages and Stages Questionnaire II total score outcomes at 4 and 12 months of age

	Prelabour CS Mean (SD)	n	Vaginal Delivery Mean (SD)	n	***P*** value	4 months	12 months
						ASQ-II total score, Spearman ρ ***(P*** value)	
**Maternal characteristics** (at admission to antenatal care), mean (SD)							
Age	33.2 (5.4)	72	31.3 (4.4)	365	0.002	−0.113 (0.02)	
Body Mass Index (kg/m^2^)	25.2 (4.2)	62	23.8 (3.8)	314	0.03		
**Infant Characteristics** mean (SD)							
Gestational Age (weeks)	38.9 (0.8)	72	40.1 (1.1)	365	< 0.001	0.243 (< 0.001)	0.107 (0.03)
Apgar score at 5 min	9.9 (0.4)	72	9.8 (0.7)	365	0.23		0.131 (0.009)
Length (cm)	50.4 (2.2)	72	50.8 (1.9)	363	0.07	0.116 (0.02)	
Birth weight (gram)	3547 (656)	72	3572 (474)	365	0.76	0.149 (0.002)	
Head circumference (cm)	35.8 (1.7)	72	34.8 (1.4)	365	< 0.001		
pH in umbilical cord artery	7.29 (0.05)	64	7.26 (0.08)	327	0.002		
Male, n (%)	34 (47.2)	72	172 (47.1)	365	> 0.99^b^		
Age at ASQ 4 months test (days)	118.4 (5.9)	66	120.7 (5.4)	352	0.002	0.226 (< 0.001)	
Age at ASQ 12 months test (days)	359.0 (7.4)	62	362.2 (9.3)	344	0.003		0.212 (< 0.001)

Abbreviations: *ASQ* Ages and Stages Questionnaire, second edition

^a^ Comparison by Students T test. ^b^ Comparison by Fischer’s exact test

**Table 2:** Predictors of selected outcomes (ASQ-II total score) at 4 and 12 months of age: results of final step of backward selection multivariate linear regression analysis

Model	ASQ-II total score 4 months			ASQ-II total score 12 months		
	n	F (***P***)	***R***^**2**^	n	F (***P***)	***R***^**2**^
	418	32.8 (< 0.001)	0.132	397	12.3 (< 0.001)	0.060
	β	95% CI	***P***	β	95% CI	***P***
**Intercept**	− 223.8	(− 341.6; −106.1)	< 0.001	− 278.9	− 484.7; − 73.1	0.008
**Gestational age**	8.1	(5.5; 10.7)	< 0.001	4.5	1.0; 8.0	0.01
**Age at test (days)**	1.3	(0.8; 1.8)	< 0.001	0.9	0.5; 1.3	< 0.001

Variables entered in model-For ASQ 4 months: maternal age, gestational age, length at birth, weight at birth, and age of infant at ASQ testing at 4 months; For ASQ 12 months: gestational age, Apgar score at 5 min, and age of infant at ASQ testing at 12 months

Gestational age was found to be strongly correlated to outcome in ASQ-II. Within the research group, we decided to not include gestational age in the regression analysis, as prelabour CS is inherently associated with an earlier gestational age.

### Funding

The study was supported by research grants from the Regional Scientific Council of Halland; the Southern Healthcare Region’s common funds for development and research; H.R.H. Crown Princess Lovisa’s Society for Childcare; and Region Skåne and the Medical Faculty, Lund University, Sweden (ALF). The funders played no role in the research process or in the writing of the paper.

## Results

Five hundred and five infants were born after prelabour CS during the study period. Of these, 98 (19%) were preterm and 34 (7%) post-term. From the 373 remaining term newborns, 174 (47%) were born after acute CS, 145 (39%) after a prelabour CS with a medical reason and 54 (14%) after a prelabour CS with no medical reason. Of the 199 prelabour term CS, 26 were not eligible due to maternal disease (diabetes *n* = 12, pre-eclampsia *n* = 6, intrauterine growth restriction *n* = 6 and combination of pre-eclampsia and IUGR *n* = 2). Furthermore, we could not include five women who smoked at admission to antenatal care, leaving 168 cases eligible for the study. Unfortunately, 96 possible cases declined participation which resulted in the inclusion of 72 deliveries with prelabour CS. The reason for declined participation was not investigated out of respect for parents’ privacy. When compared, demographic data between the study population and those who declined participation showed no significant differences in maternal age, gestational age, infants’ birth weight, length or head circumference, nor any differences in Apgar score or umbilical blood gases. We thus concluded that our study population could be regarded as representative for the entire cohort (results not shown).

Table 1 illustrates the maternal and infant characteristics of the 72 prelabour CS included in the study as compared to 365 deliveries from the vaginally delivery control-cohort. Mothers in the CS group were found to be significantly older (*P* = 0.002) and have a higher body mass index (BMI) (*P* = 0.03) as compared mothers who delivered vaginally. For infant characteristics, significant differences were found for gestational age (*P* < 0.001), head circumference (*P* < 0.001) and pH in umbilical cord artery (*P* = 0.002). No differences were found for Apgar Score at 5 min, length or birth weight between the groups. Umbilical cord clamping was performed at a mean (SD) of 32 (9) seconds in the prelabour CS group, and 89 s in the vaginal delivery group (*P* < 0.001).

ASQ-II results at 4 months for infants born after prelabour CS with cord clamping after 30 s were compared to vaginally born infants in Table 3. There was a difference in ASQ-II analysis age between prelabour CS and vaginally delivered infants. Results after adjusted analysis are presented in Table 3. Total ASQ-II score was significantly higher for vaginally born infants as compared to prelabour CS (*P* < 0.001). When analysing the subdomains of the ASQ-II, vaginally born infants were found to have higher scores for all subdomains i.e. communication, gross motor, fine motor, problem solving and personal social skills at 4 months of age (*P* < 0.05).

**Table 3:** Ages & Stages Questionnaire II results at 4 months in infants with prelabour caesarean section (CS) and cord clamping after 30 s or vaginal delivery

	Mode of delivery		Unadjusted analysis		Adjusted analysis ^a^	
	Prelabour CS *N* = 66	Vaginal Delivery *N* = 352	Mean difference (95% CI)	***P*** value ^b^	Mean difference (95% CI)	***P*** value ^b^
**Total score**	234.9 ± 41.4	258.3 ± 28.3	−23.4 (− 34.0 to − 12.8)	< 0.001	−20.7 (− 28.7 to − 12.6)	< 0.001
Communication	47.5 ± 9.3	50.3 ± 7.6	− 2.8 (− 5.3 to - 0.4)	0.02	−2.6 (− 4.7 to − 0.5)	0.02
Gross Motor	50.2 ± 10.6	54.7 ± 7.3	− 4.5 (−7.2 to − 1.8)	0.001	− 4.1 (−6.2 to − 2.0)	< 0.001
Fine Motor	41.5 ± 13.7	48.4 ± 10.5	−6.9 (− 10.4 to − 3.4)	< 0.001	−6.0 (− 8.9 to − 3.1)	< 0.001
Problem solving	49.7 ± 12.1	54.4 ± 7.7	− 4.7 (− 7.3 to − 2.2)	0.001	− 4.1 (− 6.2 to − 2.0)	< 0.001
Personal social	46.0 ± 12.1	50.5 ± 8.8	− 4.4 (− 7.5 to − 1.3)	0.006	− 4.0 (− 6.4 to − 1.5)	0.002

^a^ Adjusted for Age at testing. ^b^ Comparison by Students T test

Table 4 illustrates the ASQ-II results at 12 months of age. Total ASQ-II score was significantly higher for vaginally born infants as compared to prelabour CS (*P* = 0.04). Sub-analysis showed that the significant differences in gross motor and fine motor skills remained. After adjusting for age at testing, infants born after prelabour CS had significantly lower scores in the gross motor domain. We found no differences in communication, problem solving and personal social skills between the groups at 12 months of age.

**Table 4:** Ages & Stages Questionnaire II results at 12 months of age in infants born with prelabour caesarean section (CS) and cord clamping at 30 s or vaginal delivery

	Mode of delivery		Unadjusted analysis		Adjusted analysis ^a^	
	Prelabour CS *N* = 62	Vaginal Delivery *N* = 336	Mean difference (95% CI)	***P***-value ^b^	Mean difference (95% CI)	***P***-value ^b^
**Total score**	220.2 ± 40.9	231.3 ± 39.5	− 11.1 (− 22.9 to − 0.3)	0.04	− 8.5 (− 19.2 to 2.2)	0.12
Communication	39.8 ± 13.7	40.5 ± 12.4	−0.7 (− 4.1 to 2.8)	0.70	− 0.2 (− 3.6 to 3.3)	0.92
Gross Motor	42.3 ± 14.8	47.4 ± 14.9	−5.1 (− 9.1 to − 1.0)	0.01	−4.7 (− 8.8 to − 0.7)	0.02
Fine Motor	49.7 ± 10.1	52.2 ± 8.2	−2.5 (− 4.8. to − 0.2)	0.03	−2.0 (− 4.3 to 0.3)	0.09
Problem solving	45.7 ± 11.7	47.2 ± 11.5	− 1.5 (− 4.6 to 1.7)	0.37	−0.8 (− 3.9 to 2.3)	0.63
Personal social	42.6 ± 11.4	43.9 ± 11.7	−1.3 (− 4.5 to 1.9)	0.42	− 0.9 (− 4.1 to 2.3)	0.59

^a^ Adjusted for age at testing. ^b^ Comparison by Students T test

Although not having seen any differences in ASQ-II total score in the original cord clamping study, for transparency, we performed analyses by dividing the vaginally delivered infants into early CC (≤ 30 s) or delayed CC (≥180 s). As can be seen, the results were almost identical to when we merged vaginal deliveries in to one group except that gross motor sub-domain at 12 months was not significantly different in prelabour CS vs delayed CC after vaginal delivery (Supplementary Table 1).

In supplementary Table 2 and 3, we present adjusted ASQ-II results after adjusting for gestational age, although preterm labour is inherently associated with lower gestational age. At 4 months, all domains remained higher in the vaginally delivered group, except for communication, while no differences between the groups was found at 12 months of age.

## Discussion

We evaluated the short-term developmental outcomes of infants born with prelabour CS after a delay in cord clamping of 30 s and compared them to an historical group of vaginally born infants. Using the validated Ages and Stages Questionnaire-II, we found that infants delivered by prelabour CS had significantly lower scores as compared to vaginally born infants.

Rising caesarean rates have fuelled the current debate on the short-term and long-term health consequences of operative delivery for both the mother and newborn [23]. Such high rates cannot be explained by increases in obstetric risk factors, like post-term pregnancy, multiple infant pregnancy or maternal obesity [24–26]. Instead, this increase is believed to stem from changes in clinical practice and increased maternal request for planned CS [27, 28]. Polidano et al. [8] detailed the long-term negative cognitive outcomes of school children born by caesarean section after controlling for a large range of confounders and found that children born by CS performed a tenth of a standard deviation below in the national test scores for numeracy as compared to vaginally born children. Similar findings were also observed in a large, population-based Swedish study [9]. There is concern that many caesareans are performed without ample consideration of the consequences for both the infant and the mother [29].

Three well-known hypotheses have been put forward to explain the plausible negative consequences of prelabour CS [30]. The maternal microbiome hypothesis takes in to consideration the role of reduced maternal vaginal flora exposure in infants born by prelabour CS. Alterations in infant immunological development have been proposed to last from a few weeks to several years after birth [31–33]. The second hypothesis considers the impact of reduced mechanical forces and stress hormones with CS. These have been implicated in triggering important developmental cues for the fetus in preparation for extrauterine life [30]. Lastly, the third hypothesis deals with possible epigenetic modifications of gene expression as a result of “artificial labour” and imply alterations in DNA methylation in certain cases. However, there is still inadequate data to fully support this hypothesis [23].

The findings of our study show that vaginally born infants have improved neurodevelopmental scores as compared to surgically born infants already at 4 months of age. These differences were apparent in all domains of ASQ-II scoring and continued for gross motor skills at 12 months of age. These differences remained regardless of cord clamping time (early CC ≤ 30 s vs delayed CC ≥ 180 s) in vaginal deliveries at 4 months but not at 12 months. Nevertheless, these findings would indicate that the timing of cord clamping in vaginal deliveries, which is a proxy for placental transfusion, was not a factor that accounted for the neurodevelopmental changes observed in the study. This can be explained by differences in the physiology of placental transfusion in prelabour CS since a faster placental transfusion can be secondary to a lower blood pressure due to less circulating adenosine and catecholamines [34, 35] as compared to vaginally born infants.

Our data adds to the growing body of evidence that advocates for caution against the increasing rates of CS globally. If caesarean section is planned before the natural onset of labour, the baby is commonly delivered at an earlier gestational age. In our study, the difference was 1.2 weeks, and a portion of the differences in ASQ scores may be explained by this difference, as shown in Supplementary Tables 2 and 3. These findings suggest that obstetricians should be encouraged to plan caesarean deliveries as close to 40 + 0 weeks as possible. Further studies are indicated to explore the optimal time-point for planned caesarean deliveries, in order to avoid an increased risk for the spontaneous onset of labour in the group.

Placental transfusion for 30 s with prelabour CS has been associated with comparable 4 month infant iron stores after 180 s of delayed CC with vaginal birth [13]. However, our study showed that this was not correlated to a corresponding neurodevelopmental assessment score in infants born with prelabour CS. This may indicate that physiological mechanisms related to factors other than iron stores can be responsible for differences in neurodevelopment in this group of infants.

The small sample size in the CS group can be considered as one of the main limitations of the current study. The infants were also compared to a historical control group, which could have an effect on the internal validity of the study, in combination with the risk of selection bias due to the inclusion rate of 72 out of 168 eligible cases. Unidentified differences in baseline characteristics between groups, including prenatal maternal as well as peri- and postnatal infant influences may contribute to bias. Two main confounders were identified, age at testing and gestational age. The first was adjusted for and affected the findings of the current study only to a minor degree, whilst adjusting for gestational age affected the neurodevelopmental results mainly at the 12 months assessment. For external validity, we presented outcome adjusted values for gestational age as a supplement, as obstetricians are required to perform prelabour CS at an earlier gestational age to avoid the spontaneous start of labour in pregnant women. However, even after taking these limitations into account, the results of this study indicate lower neurodevelopmental scores as assessed by the ASQ-II during infancy, in particular at 4 months of age, after prelabour CS as compared to vaginal delivery.

## Conclusions

We compared the 4 and 12 month neurodevelopmental outcome of a group of infants born with prelabour CS to a historical group of vaginally born infants using the validated Ages and Stages Questionnaire-II. We found that infants delivered by prelabour CS had significantly lower scores in all developmental domains at the 4 month evaluation as compared to vaginally born infants and that these differences remained for the gross-motor skills domain at the 12 month assessment. This may indicate that negative differences in neurodevelopmental outcome in infants born by prelabour CS may be apparent already a few months after birth. In combination with recent reports on affected assessments in school-going children, the results of this study suggest that obstetricians should take these risks into consideration when discussing the consequences of prelabour CS with their patients.

## Supplementary information

**Additional file 1: Table S1.** Results from the Ages and Stages Questionnaire-II (ASQ-II) at 4 and 12 months of age in infants born with prelabour caesarean section (CS) and cord clamping at 30 s compared to vaginal delivery with early cord clamping (≤ 30 s) or delayed cord clamping (≥ 180 s). **Table S2.** Results from ASQ-II at 4 months in infants with prelabour CS and cord clamping after 30 s or vaginal delivery. **Table S3.** Results from ASQ-II at 12 months of age in infants born with prelabour CS and cord clamping at 30 s or vaginal delivery.

## Data Availability

The datasets used and/or analysed during the current study are available from the corresponding author on reasonable request.

## Abbreviations

ASQ-II: Ages and Stages Questionnaire, second edition
CS: Caesarean section
CC: Cord clamping
SD: Standard Deviation
