# Supplementary material for: Prelabour caesarean section and neurodevelopmental outcome at 4 and 12 months of age: an observational study
Source: BMC Pregnancy Childbirth. 2020 Sep 25;20:564. doi: 10.1186/s12884-020-03253-8 (PMC7517619; doi:10.1186/s12884-020-03253-8)
Supplement: Supplementary file 1 — Additional file 1: Table S1. Results from the Ages and Stages Questionnaire-II (ASQ-II) at 4 and 12 months of age in infants born with prelabour caesarean section (CS) and cord clamping at 30 s compared to vaginal delivery with early cord clamping (≤ 30 s) or delayed cord clamping (≥ 180 s). Table S2. Results from ASQ-II at 4 months in infants with prelabour CS and cord clamping after 30 s or vaginal delivery. Table S3. Results from ASQ-II at 12 months of age in infants born with prelabour CS and cord clamping at 30 s or vaginal delivery. [file 12884_2020_3253_MOESM1_ESM.docx]

**Supplement Table 1.**

Results from the Ages and Stages Questionnaire-II (ASQ-II) at 4 and 12 months of age in infants born with prelabour caesarean section (CS) and cord clamping at 30 seconds compared to vaginal delivery with early cord clamping (≤ 30 s) or delayed cord clamping (≥ 180 s)

|  | | **Prelabour CS** | **Cord clamping ≤ 30 s** | | | **Cord clamping ≥ 180 s** | | |
| --- | --- | --- | --- | --- | --- | --- | --- | --- |
|  |  | Mean (SD) | Mean (SD) | Mean difference  (95% CI) | *P* value | Mean (SD) | Mean difference  (95% CI) | *P* value |
| **ASQ-II 4 months** | | N=66 | N=175 |  |  | N=177 |  |  |
|  | **Total score** | 234.9 (41.4) | 257.2 (29.2) | -22.3 (-33.0 to -11.6) | <0.001 | 259.4 (27.5) | -24.5 (-35.2 to -13.8) | <0.001 |
|  | Communication | 47.5 (9.3) | 50.1 (7.6) | -2.6 (-5.3 to 0.2) | 0.07 | 50.6 (7.7) | -3.1 (-5.8 to - 0.3) | 0.02 |
|  | Gross Motor | 50.2 (10.6) | 54.3 (7.7) | -4.1 (-6.8 to -1.3) | 0.001 | 55.1 (6.8) | -4.9 (-7.6 to -2.2) | <0.001 |
|  | Fine Motor | 41.5 (13.7) | 47.7 (11.4) | -6.3 (-10.1 to -2.4) | <0.001 | 49.0 (9.6) | -7.5 (-11.4 to -3.7) | <0.001 |
|  | Problem solving | 49.7 (12.1) | 53.5 (8.3) | -3.8 (-6.6 to -1.0) | 0.004 | 55.4 (7.0) | -5.7 (-8.5 to -2.9) | <0.001 |
|  | Personal social | 46.0 (12.1) | 51.6 (8.2) | -5.6 (-8.8 to -2.3) | <0.001 | 49.4 (9.5) | -3.3 (-6.6 to -0.1) | 0.04 |
| **ASQ-II 12 months** | | N=62 | N=166 |  |  | N=170 |  |  |
|  | **Total score** | 220.2 (40.9) | 233.1 (38.3) | -12.8 (-27.1 to 1.3) | 0.09 | 229.6 (40.6) | -9.3 (-23.5 to 4.8) | 0.34 |
|  | Communication | 39.8 (13.7) | 40.5 (12.2) | -0.6 (-5.2 to 3.9) | >0.99 | 40.5 (12.7) | -0.7 (-5.2 to 3.8) | >0.99 |
|  | Gross Motor | 42.3 (14.8) | 48.4 (14.3) | -6.0 (-11.4 to -0.7) | 0.02 | 46.5 (15.5) | -4.2 (-9.5 to 1.1) | 0.18 |
|  | Fine Motor | 49.7 (10.1) | 52.3 (8.3) | -2.6 (-5.7. to 0.4) | 0.12 | 52.1 (8.1) | -2.5 (-5.5. to 0.6) | 0.16 |
|  | Problem solving | 45.7 (11.7) | 47.6 (11.2) | -1.9 (-6.1 to 2.2) | 0.79 | 46.7 (11.8) | -1.0 (-5.2 to 3.1) | >0.99 |
|  | Personal social | 42.6 (11.4) | 44.3 (11.0) | -1.6 (-5.8 to 2.5) | >0.99 | 43.6 (12.4) | -1.0 (-5.2 to 3.2) | >0.99 |

**Supplement Table 2**.

Results from ASQ-II at 4 months in infants with prelabour CS and cord clamping after 30 seconds or vaginal delivery

|  | **Mode of delivery** | | **Unadjusted analysis** | | **Adjusted analysis** ^a^ | |
| --- | --- | --- | --- | --- | --- | --- |
|  | **Prelabour CS**  N=66 | **Vaginal Delivery**  N=352 | **Mean difference**  **(95% CI)** | ***P* value** | **Mean difference**  **(95% CI)** | ***P* value** |
| **Total score** | 234.9 (41.4) | 258.3 (28.3) | -23.4 (-34.0 to -12.8) | <0.001 | -13.0 (-21.5 to -4.5) | 0.003 |
| Communication | 47.5 (9.3) | 50.3 (7.6) | -2.8 (-5.3 to - 0.4) | 0.02 | -2.0 (-4.3 to 0.3) | 0.09 |
| Gross Motor | 50.2 (0.6) | 54.7 (7.3) | -4.5 (-7.2 to -1.8) | 0.001 | -2.6 (-4.8 to -0.3) | 0.03 |
| Fine Motor | 41.5 (13.7) | 48.4 (10.5) | -6.9 (-10.4 to -3.4) | <0.001 | -3.3 (-6.4 to -0.2) | 0.03 |
| Problem solving | 49.7 (12.1) | 54.4 (7.7) | -4.7 (-7.3 to -2.2) | 0.001 | -2.3 (-4.5 to 0.0) | 0.05 |
| Personal social | 46.0 (12.1) | 50.5 (8.8) | -4.4 (-7.5 to -1.3) | 0.006 | -2.8 (-5.5 to -1.5) | 0.04 |
| ^a^ Adjusted for gestational age and age at testing | | | | | | |

**Supplement Table 3.**

Results from ASQ-II at 12 months of age in infants born with prelabour CS and cord clamping at 30 seconds or vaginal delivery

|  | **Mode of delivery** | | **Unadjusted analysis** | | **Adjusted analysis**^a^ | |
| --- | --- | --- | --- | --- | --- | --- |
|  | **Prelabour CS**  N=62 | **Vaginal Delivery**  N=336 | **Mean difference**  **(95% CI)** | ***P*-value** | **Mean difference**  **(95% CI)** | ***P*-value** |
| **Total score** | 220.2 (40.9) | 231.3 (39.5) | -11.1 (-22.9 to -0.3) | 0.04 | -3.8 (-15.3 to 7.7) | 0.52 |
| Communication | 39.8 (13.7) | 40.5 (12.4) | -0.7 (-4.1 to 2.8) | 0.70 | 0.2 (-3.5 to 4.0) | 0.90 |
| Gross Motor | 42.3 (14.8) | 47.4 (14.9) | -5.1 (-9.1 to -1.0) | 0.01 | -3.2 (-7.6 to 1.2) | 0.15 |
| Fine Motor | 49.7 (10.1) | 52.2 (8.2) | -2.5 (-4.8. to -0.2) | 0.03 | -1.0 (-3.4 to 1.5) | 0.45 |
| Problem solving | 45.7 (11.7) | 47.2 (11.5) | -1.5 (-4.6 to 1.7) | 0.37 | -0.6 (-4.0 to 2.7) | 0.73 |
| Personal social | 42.6 (11.4) | 43.9 (11.7) | -1.3 (-4.5 to 1.9) | 0.42 | 0.7 (-2.7 to 4.2) | 0.67 |
| ^a^ Adjusted for gestational age and age at testing | | | | | | |
